# 

**Published:** 1846-06

**Authors:** 


					﻿JUNE AND JULY, 1846.
VOL. I.
NEW SERIES.
NO. II.
ILLINOIS AND INDIANA
MEDICAL AND SURGICAL
JOURNAL.
K
E-i
£
O
s
ci
a
S
H
O
fx
Pi
a
t>
a
p
a
K
a
M
a
a
a
a
td
a
g
w
td
a
o
o
H
M
HI
iz|
p
2
H
td
H
kJ
i
tc
M
>
P
td
w
EDITED BY
JAMES V. Z. BLANEY, M. D.
DANIEL BRAINARD, M. D.
WM. B. HERRICK, M. D., and
JOHN EVANS, M. D.
CHICAGO, ILLS.:
PUBLISHED BY ELLIS & FERGUS
INDIANAPOLIS, IND.: ,
PUBLISHED BY C. B. DAVIS.
1846.
TWO DOLLARS PER ANNUM, IN ADVANCE.
				

